# The Feasibility and Effectiveness of a Novel, On-Line Social Skills Intervention for Individuals With Prader-Willi Syndrome

**DOI:** 10.3389/fpsyt.2022.863999

**Published:** 2022-05-24

**Authors:** Elisabeth M. Dykens, Elizabeth Roof, Hailee Hunt-Hawkins, Charles McDonald

**Affiliations:** ^1^Department of Psychology and Human Development and Vanderbilt Kennedy Center, Vanderbilt University, Nashville, TN, United States; ^2^Psychiatric Nursing Program, Vanderbilt University School of Nursing, Nashville, TN, United States

**Keywords:** building social skills in Prader-Willi syndrome, social cognition, social isolation, loneliness, COVID-19, telemedicine

## Abstract

**Introduction:**

People with neurodevelopmental disabilities, including Prader-Willi syndrome (PWS), are at heightened risk for the negative sequalae of loneliness, including depression and anxiety. While societal factors such as stigma or limited social opportunities contribute to loneliness, so too do deficits in social cognition and social skills. People with PWS have specific difficulties recognizing affect in others, accurately interpreting social interactions, and taking the perspectives of others. These features, combined with hyperphagia, rigidity, and insistence on sameness conspire to impede the abilities of people with PWS to make and sustain friendships and reduce feelings of loneliness.

**Methods:**

We developed and administered an intervention, Building Our Social Skills (BOSS), that aimed to improve social skill deficits in PWS. The 10-week intervention was administered on-line via Zoom to 51 young people with PWS in the U.S. (M age = 20.8, SD = 6.42). Two clinicians co-led groups of 6–8 participants in 30-min sessions, 3 times per week, and also trained 4 graduate students to co-lead groups with high fidelity. We used a pre-post intervention and 3-month follow-up design, with no control group, and mitigated this design limitation by triangulating across informants and methodologies. Specifically, parents completed the widely used Social Responsiveness Scale (SRS) and Child Behavior Checklist (CBCL), and participants were individually interviewed about their friendships and loneliness. Interview responses were reliably coded by independent raters.

**Results:**

Repeated measure multivariate analyses, with baseline values entered as covariates, revealed significant pre-to post-test improvements in the SRS's social cognition, motivation and communication subscales (*p*'s < 0.001), with large effect sizes (${\text{n}}_{\text{p}}^{2}$ = 0.920, 0.270, and 0.204, respectively). Participant and parental reports of loneliness were correlated with the CBCL's Internalizing domain, specifically the Anxiety/Depressed subdomain. Over time, parents reported getting along better with peers, increased contact with friends, more friends and less loneliness. Participants also reported significantly less loneliness and more friends.

**Conclusions:**

This mixed method, proof-of-concept study demonstrated the feasibility of delivering an on-line social skills intervention to young people with PWS. As no differences were found between clinician vs. graduate student outcomes, the BOSS curriculum holds considerable promise for wider dissemination and implementation in the PWS community.

“It is a good thing to have many friends. No one would choose to live without friends even if he possessed all other goods…True friends wish the good of each other.”

Aristotle, Nicomachean Ethics, 349 BC

## Introduction

Aristotle foretold what contemporary studies in psychology, sociology and social neuroscience have collectively confirmed: that the human brain is wired for social engagement and that friendships and connections to others are the cornerstones of human flourishing and well-being (1, 2). Yet some people struggle to develop friends and are at risk for social isolation or loneliness, especially those with intellectual disabilities (ID). Both societal factors and characteristics of ID hinder the ability of these individuals to successfully engage with others and to develop and maintain friendships (3).

At the societal level, people with ID often experience stigma, discrimination and restricted opportunities for community engagement (3). Relative to the general population, individuals with ID also experience elevated rates of bullying and abuse (4). At the same time, ID is defined by cognitive and adaptive deficits in conceptual, practical and social domains (5). Individuals may, for example, have problems communicating and taking the perspectives of others, as well as with performing such executive functions as focusing, attending to and evaluating pertinent stimuli, planning, self-regulating and controlling emotions (6, 7). Deficits in any these areas are likely to impede optimal social functioning.

Taken together, these societal forces and attributes of ID contribute to the increased rates of loneliness and social isolation in this population. Loneliness and social isolation are related but distinct concepts. Loneliness is conceptualized as a negative emotional response to the discrepancy between one's actual vs. desired quantity or quality of social interactions (8). In contrast, social isolation is an objective index that quantifies one's social contacts, often assessed through social network size. While isolation is a risk factor for loneliness, even those with enriched social networks may still feel lonely. Further, those who are isolated may be content with their solitude, without experiencing loneliness.

Both loneliness and social isolation have been studied in people with ID. Macdonald et al. (9) found that a full 73% of 310 individuals with cognitive impairments or other developmental disabilities indicated that they were lonely. In a review of studies that sampled over 11,000 adults with ID, Alexandra et al. (10) calculated an average loneliness prevalence rate of 44.7%. Despite variability in rates across these studies, people with disabilities experience loneliness to a greater degree than the general population (11). Further, loneliness in people with ID is associated with poor mental health, especially depression (10, 12).

Loneliness or social isolation may intensify in certain developmental periods. For example, once young adults with ID leave formal schooling, they are at heightened risk for social isolation or loneliness, as they have lost the built-in social connections, supports, and services that schools provide. Navigating the fragmented adult service system in the U.S. is challenging, and many adults with ID lack employment or meaningful social and recreational opportunities. As such, compared to others, adults with ID have fewer friendships and smaller social networks that are often limited to family members or paid care providers (13–16). Asselt-Goverts and colleagues (17), however, reported that the majority (73%) of their participants with ID were satisfied with the size of their social networks. Instead, they expressed desires to strengthen their existing relationships, as well as to bolster their skills interacting with others.

Although studies to date have focused on ID in general, people with Prader-Willi syndrome (PWS) have several phenotypic features that place them at even higher risk for loneliness or social isolation. PWS is a genetic, neurodevelopmental disorder caused by the lack of paternally imprinted genes on chromosome 15q11–15q13, either through paternal deletions that vary in size or through maternal uniparental disomy (mUPD), or when both copies of chromosome 15 are maternally inherited (18). Hyperphagia, often cast as the hallmark of this syndrome, begins in early childhood and is associated with aberrant neural networks involved in satiety and reward. Impaired satiety results in a state in which individuals are habitually hungry yet rarely feel full (19–21). People with PWS thus need external dietary controls and constant food supervision to avoid becoming morbidly obese (18). And, as food is readily available in most communities or family social gatherings, hyperphagia also restricts opportunities for engaging with others.

PWS is also characterized by mild to moderate intellectual disability, growth hormone deficiencies, temper outbursts, rigidity, insistence on sameness, and repetitive, compulsive behaviors (22–26). While approximately 12.3% of individuals with PWS have co-occurring autism spectrum disorder, many more show some degree of impairment in the quality or quantity of their reciprocal communication (24). Further, people with PWS often exhibit executive function difficulties, especially with attention and task switching (27). Many individuals thus have difficulties modifying their behavior to fit changes or nuances in social interactions and may instead respond to social situations with temper outbursts, impulsivity, and rigid thinking (27).

Given these phenotypic features, people with PWS often have significant problems sustaining friendships and getting along with peers or others (22, 24, 28). Such interpersonal problems are also associated with deficits in social cognition, or those processes that enable people to understand and successfully engage in the social world (29). These processes include recognizing emotional states in others, understanding what others are thinking (theory of mind), and using social cues to draw inferences about interpersonal or social situations (social perception). People with PWS often show deficits in these key relationship-building skills.

First, they have difficulties recognizing affect in others. Consistent across two studies (30, 31), participants with PWS readily identified happy, and were significantly better at identifying anger than sadness or fear. Examining affect recognition over a 2-year time period in 94 individuals with PWS, Dykens et al. (30) found that participants improved in their recognition of fear, but no significant gains were found for sadness. Further, even with some improvement, recognition of these negative emotions remained at chance levels, and sad was often mistaken for anger, and anger for sad.

Second, people with PWS show impairments in high-order theory of mind tasks. Administering false-belief theory of mind tasks to 66 children and youth with PWS, Lo, and colleagues (32) found that participants generally understood another person's mistaken belief, or so-called first order-beliefs. Similarly, Tager-Flusberg and Sullivan (33) reported that 10 children with PWS outperformed those with Williams syndrome on a first-order false belief task. Lo et al. (32), however, found that people with PWS struggled with more complex second-order tasks, or identifying what one person thinks about another person's beliefs.

Finally, just one study has examined the social perceptions of people with PWS, or how they use social cues to interpret interactions between people. Dykens et al. (31) longitudinally administered videotaped social perception vignettes to 94 individuals with PWS that depicted negative events with either sincere/benign or insincere/hostile interactions between peers. Participants made some gains over time detecting pertinent social cues, but not in using these cues to form correct conclusions about the intentions of others. They had consistent difficulties in accurately judging the sincere intentions of others, but over time performed better in correctly judging interactions involving trickery, deceit and lying.

Given such findings, interventions are sorely needed to improve social functioning in people with PWS. Group interventions teaching social engagement and communication skills have been deemed an evidence-based practice in individuals with autism spectrum disorder (34–36) and in other groups with impaired social skills [e.g., schizophrenia, (37–39)]. Yet no such interventions have yet been tried in people with PWS.

The first aim of this study, then, was to test the practicality and tolerability of a novel, on-line, group intervention aimed at improving social cognition and social engagement skills in adolescents and adults with PWS. Demonstrating feasibility was especially important as the intervention required a significant time commitment from both participants and group leaders; 30-min sessions were conducted 3 times a week for 10 consecutive weeks.

Our second aim was to determine how well participants with PWS responded to the intervention. We hypothesized that, on average, participants would show less social dysfunction but improved social engagement skills. We further expected that these gains would be associated such real-word outcomes as an increased number of friends, getting along better with others, and more sophisticated understandings of what friendships mean. We also hypothesized that loneliness would be associated with internalizing symptoms, and that feelings of loneliness as reported by both participants and parents would diminish over time.

## Methods

### Design

As a proof-of-concept study, we used a quantitative and qualitative, mixed-method, pre-post intervention and 3-month follow-up design, with no control group. We mitigated the limitations of this quasi-experimental design by triangulating across informants and methodologies (38–40). Specifically, baseline, post-intervention and 3-month follow-up data were obtained from two sources: parents completed standardized measures of social and behavioral functioning; and participants with PWS were administered semi-structured interviews regarding their friendships and loneliness. Although individual differences are often found between informant ratings of emotions or behaviors (41), triangulation increases the credibility of a study if similar findings are obtained from different informants and methodologies.

### Participants

The intervention included 51 adolescents or young adults with genetically confirmed PWS aged 14 to 33 years who resided in the U.S. (M age = 20.82 years, SD = 4.63; 45.1% male). The study was posted via PWS-related social media outlets and included the need for prospective participants to have access to a computer and the internet. As shown in Table 1, participants varied in PWS genetic subtypes, with most (70.6%) having paternal deletions. We aimed to recruit participants both in and out of high school to determine if school status was associated with dependent measures or outcomes. Approximately half were still in high school (*n* = 24), and of the 27 high school graduates, just 5 were employed.

**Table 1:** Demographic variables for 51 participants with PWS and their parents.

	**M (SD) or %**	
**PWS demographics**		
Age (M, SD)	M = 20.82 (4.63)	
Age range	14 to 33 years	
Male	45.1%	
Female	54.9%	
White	92%	
Black or asian	8%	
In high school	47.1%	
Graduated high school	52.9%	
Employed graduates	18%	
Living in family home	88%	
Living outside family home	12%	
Paternal deletion	70.6%	
mUPD	25.5%	
Imprinting defect	3.9%	
**Parent demographics**		
Maternal age	M = 51.01 (6.53)	
Paternal age	M = 53.33 (8.67)	
Education	Maternal	Paternal
High school	16.6%	37.5%
2-Year college	9.8%	8.3%
4-Year college	41.2%	20.8%
Professional/Graduate	32.4%	33.3%

A power analysis was conducted using rates of loneliness in a separate population of individuals with PWS and anticipated rates of loneliness in our participants post-intervention. Setting the alpha at 0.05 and power at 0.80 yielded a sample size of 48. We over recruited as we anticipated some attrition. Indeed, an additional eight individuals were enrolled but then withdrew from the study either after baseline or in the first few weeks of the intervention. Reasons for withdrawal included scheduling conflicts, disruptive behaviors during sessions and/or an unwillingness to participate in the curriculum. No significant differences emerged between completers vs. non-completers in age, gender, genetic subtypes or baseline scores on dependent variables.

### Procedures

#### Consent

The study was approved by the Behavioral Science Institutional Review Board (IRB) at Vanderbilt University (IRB# 16155). Consistent with IRB procedures, parents provided written, informed consent while individuals with PWS provided written, informed assent. We ensured that participants and their parents understood the time commitments involved in the study as well as the need for sessions to be recorded for training and research purposes.

#### Group Sessions and Leaders

Six to eight participants were enrolled in 30-min group sessions that met 3 times per week for 10 consecutive weeks via Zoom. To avoid scheduling conflicts and facilitate compliance, sessions were scheduled at the same time each week. All sessions were recorded.

Two clinicians with expertise in PWS co-led 46% of the group sessions. To mitigate “therapist effects,” or the possibility that some interventionists consistently achieve superior outcomes than others (42), the two clinicians trained and supervised four graduate students (who had minimal exposure to PWS) to co-lead the remaining 54% of sessions. Students were supervised 2–3 times a week until they became comfortable with the curriculum and managing participants. Students then met with the supervising clinicians once-weekly or on an as-needed basis.

The clinical supervisors ensured high treatment fidelity by reviewing at least one graduate student led taped session weekly using well-accepted criteria (43). These included preparing materials for sessions; establishing rapport and group rules; adhering to curriculum lessons and content; appropriately engaging participants; reviewing content; and encouraging participants to practice specific lessons outside of group time.

#### BOSS Curriculum

**Figure 1:**
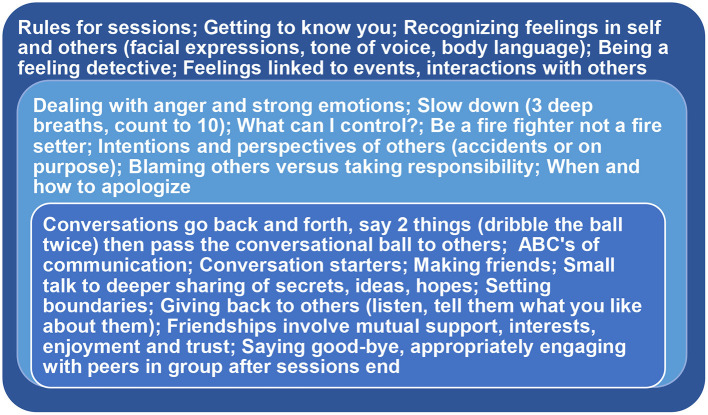
Major topics addressed in the BOSS curriculum. Beginning with basic emotion recognition skills in one's self and others, the curriculum then emphasized regulating one's own emotions in order to accurately perceive the intentions and perspectives of others. Building on these skills, the program ended with lessons about conversing, listening and developing and maintaining friendships.

The curriculum was based on the social skills deficits typically encountered in PWS. It was divided into three modules that functioned synergistically, with each module building on previous lessons. As shown in Figure 1, the curriculum began with teaching such basic social cognition skills as recognizing emotional expressions in others, taking another person's point of view, and correctly interpreting the intentions of others. The second module focused on recognizing affect in one's self, especially such strong negative emotions as anger, and how to best handle them via self-control, apologizing and taking responsibility. The curriculum ended with a module on making friends and such social engagement skills as starting a conversation, conversational turn-taking, listening to others, giving back, and moving from superficial exchanges to trusting one another and a deeper sharing of thoughts.

For one session toward the end of each module, participants were given an exercise to practice together based on lessons learned, without being guided by group leaders. Group leaders remained on Zoom to observe, provide feedback to individuals at the next session and assist as needed.

Participants were encouraged to practice specific skills at home or in the community, and to bring their experiences doing so to the next session. Parents were provided with brief descriptions of each week's curriculum, giving them the option of reinforcing concepts or practicing skills with their child.

### Measures

#### Demographics

Parents completed a brief questionnaire regarding their child's age, gender, genetic subtype of PWS, and previous or current schooling and employment status. Parental age and educational status were also ascertained for descriptive purposes of the sample.

#### Social Responsiveness Scale-2

Parents completed this 64-item questionnaire (44) that assesses social impairments often seen in autism and other developmental disabilities. Items were rated using a 1 to 4 scale; 1 = Not true, 2 = Sometimes true, 3 = Often true, 4 = Almost always true. Seventeen items are reverse scored.

The SRS includes four social subscales and a repetitive and restricted behavior subscale. As the intervention did not target repetitive behaviors, this subscale was not included in analyses. The social subscales include: Social Cognition, 12 items that tap the ability to interpret social behavior (Cronbach's alpha = 0.75); Social Communication, 22 items that assess reciprocity in social interactions (Cronbach's alpha = 0.87); Social Motivation, 11 items that tap the extent to which individuals are motivated to engage and interact with others (Cronbach's alpha = 0.80); and Social Awareness, 8 items that measure social cue recognition. Unlike the other subscales, however, the Cronbach's alpha for the Awareness subscale was unacceptable (45) and eliminating two infrequently endorsed items did not improve the alpha. As such, we did not include this subscale in analyses. As recommended by Constantino and Gruber (44), in order to detect changes in social functioning analyses used raw rather than standardized scores. Higher scores indicate more symptoms.

#### Child Behavior Checklist

The CBCL (46) is a 113-item checklist of internalizing and externalizing problems completed by parents using a 3-point scale, 0 = not true; 1 = somewhat or sometimes true; 2 = very true or often true. In light of previous work on loneliness and internalizing problems, the study only used the Internalizing Problems Domain (Cronbach's alpha = 0.79), which consists of three subdomains (Anxious/Depressed, Depressed/Withdrawn and Somatic Complaints). Domain raw scores were used in correlational analyses; higher scores indicate more problems. The study also analyzed one question from a separate CBCL subdomain (Social Problems), specifically “Complains of feeling lonely.”

The CBCL also includes a social competence domain. Three questions were analyzed from this domain that probed patterns of social interactions: (1) Aside from family members, how many close friends does your child have? (scored 0 = 0–1 friend, 1= 2–3 friends, 2 = 4 or more friends); (2) How often does your child visit friends outside of school/work time (scored 0 = < once a week, 1 = 2–3 times per week, 2 = 3 or > times per week); and (3) Compared to others his/her age, how does your child get along with family members and peers, and how well do they work or play alone (scored 0 = worse, 1 = average, 2 = better).

#### Semi-structured Interviews

Participants were individually interviewed by two graduate students who did not serve as group leaders. The interviews provided a structure for gathering data while also allowing interviewers to clarify or follow-up on comments. Interviews took from 10 to 15 min to complete and were audiotaped for subsequent transcription.

After introductions and rapport building, participants were asked about their friendships and feelings of loneliness. Starting with a general probe, “Tell me about your friends”, interviewers followed up with specific queries: “How many friends do you have?”, “What are their names?”, “Where did you meet them?”, “What do you like to do for fun with your friends?” and “What does being a friend mean to you?” or “How would you describe a friend?” Participants were also asked if they ever felt lonely and if so, if there were things that they do to help them feel less lonely.

Consistent with procedures for emergent content coding (47), transcribed interviews were read several times by two members of the research team in order to develop codes that best captured participants' responses. Most responses fell into objective or straightforward coding categories. Responses to the loneliness question, for example, were captured by codes of no, sometimes or yes. The number of friends was verified by asking for friends' names, or what they did together. Similarly, responses to where participants met their friends or what they did together were readily apparent. One question, however, involved making more subjective judgments, specifically “What does a friend mean to you?” or “How would you describe a friend?” As such, answers to this question were independently coded by two members of the research team. Inter-rater reliability was very high at the pre, post and 3-month follow-up assessments (kappa's = 0.93, 0.86, and 0.89, respectively).

As we observed that responses to this question differed in tone, the same two individuals also independently rated the valence of participants' descriptions of friends as either: positive/neutral (fun, good, like them, I have friends) vs. negative. Negatively-toned responses included a loss of contact with friends (e.g., “I used to see them but not anymore”, “They are too busy, I don't hang out with them that much”) as well as backtracking from their initial responses (e.g., “Yeah, I got friends, but maybe not much really”). Codes were based on all responses over time, and inter-rater reliability was high (kappa = 0.79).

### Statistical Analyses

Analyses included data from individuals who completed the program, without baseline values from non-completers. We justified using an “as-treated” vs. “intend-to-treat” approach as this is the first study to assess a novel intervention (48). Preliminary analyses found no significant effects of age, gender or PWS genetic subtype on dependent measures that would need to be controlled for in subsequent analyses. Similarly, we did not control for group leaders (clinicians vs. graduate students), as there were no significant leader effects in baseline or follow-up evaluations.

Repeated measure multivariate analyses were used to assess changes over time in SRS raw scores. For each analysis, the corresponding baseline score was entered as a covariate. If significant interactions with baseline scores were found, we determined if differential treatment effects were found in those with higher vs. lower baseline scores. Effect sizes were estimated by the partial eta^2^ ($\eta_{\text{p}}^{2}$) and interpreted as: η^2^ = 0.01 = a small effect; η^2^ = 0.06 a medium effect; and η^2^ = 0.14 a large effect (49).

Related Samples Cochran's Q Tests were used to assess changes over time in parent and self-reports of loneliness, number of friends and other CBCL social interaction variables. Cochran's Q, commonly used to analyze categorical longitudinal data, requires dichotomous variables. As study variables had 3 or more possible ratings, **Tables 3, 4, 7** note how data were meaningfully collapsed into 2 categories. For transparency, however, these three Tables present all ratings.

Spearman's rho correlations assessed relationships between the CBCL's Internalizing domain with parent and self-reports of loneliness. If significant, we followed up with correlations with the sub-domains subsumed under this domain.

## Results

### Practicality and Tolerability

The intervention was well-tolerated by participants. They logged onto Zoom with minimal help yet did need occasional reminders to adjust their computer screens or microphones. Reliable internet connectivity was noted to be a challenge for two participants. Group leaders observed that participants were on time, prepared and engaged. Informal feedback from parents and group leaders indicated that individuals enjoyed meetings and took them seriously. Many participants stated that they looked forward to sessions and were disappointed when the intervention ended. Ways to address their disappointment and say good-bye were directly addressed in the BOSS curriculum.

### Social Responsiveness Scale

Mauchly's Tests of Sphericity were significant for the three repeated measures ANOVAS; as such Greenhouse-Geisser corrections were applied to adjust degrees of freedom. Analyses revealed significant main effects of time for all three SRS subscales, with large effect sizes: Motivation *F*_(1.6, 81.2)_ = 18.53, *p* < 0.001, ${\text{n}}_{\text{p}}^{2}$ = 0.270; Communication *F*_(1.62, 81.26)_ = 12.84, *p* < 0.001, ${\text{n}}_{\text{p}}^{2}$ = 0.204; and Cognition *F*_(1.67, 83.57)_ = 20.41, *p* < 0.001, ${\text{n}}_{\text{p}}^{2}$ = 0.920. See Table 2 for mean scores. In all analyses, baseline scores significantly differed from the end of the intervention and from the 3-month follow-up. In the Cognition subscale, the end of intervention also differed from the 3-month follow-up.

**Table 2:** Descriptive statistics, F's and p's for significant interactions between time and baseline raw scores on the Social Responsiveness Scale subscales.

**SRS subscales**	**Baseline M (SD)**	**End of intervention M (SD)**	**3 month follow-up M (SD)**	**F, p**	** ${\text{n}}_{\text{p}}^{2}$ **
**Social cognition total mean**	25.23 (6.52)	22.76 (6.38)	21.44 (6.24)	7.13^***^	0.137
Low baseline	18.50 (2.68)	18.55 (4.11)	17.05 (4.59)		
Middle baseline	24.43 (1.31)	21.87 (3.98)	21.12 (3.98)		
High baseline	32.76 (3.42)	27.88 (6.46)	26.17 (6.00)		
**Social motivation total mean**	13.02 (5.98)	11.00 (5.45)	10.37 (4.27)	29.47^***^	0.367
Low baseline	7.31 (1.95)	7.62 (3.09)	7.68 (2.91)		
Middle baseline	12.25 (0.85)	10.62 (2.94)	9.43 (1.59)		
High baseline	19.52 (4.69)	14.78 (4.09)	14.01 (4.37)		
**Social communication total**	23.45 (7.51)	19.52 (7.79)	18.72 (7.77)	5.88^**^	0.107
Low baseline	14.38 (3.45)	12.61 (4.17)	12.31 (4.75)		
Middle baseline	21.55 (1.43)	20.90 (5.40)	19.55 (5.83)		
High baseline	31.44 (4.56)	25.05 (7.07)	24.31 (7.48)		

^**^p < 0.01; ^***^p < 0.001.

These main effects of time, however, were qualified by significant interactions with baselines scores, again with large effect sizes. Table 2 summarizes the F and ${\text{n}}_{\text{p}}^{2}$ values and for these interaction terms. To help explain these interaction effects, participants' baseline scores were used to assign them into low, middle or high scoring groups for each subscale. Follow-up repeated measures ANOVAs were conducted with groups entered as a between-subjects factor. These were significant; Social Cognition, *F*_(3.5, 84.3)_ = 4.47, *p* = 0.004, ${\text{n}}_{\text{p}}^{2}$ = 0.157; Social Motivation *F*_(3.42, 82.14)_ = 10.35, *p* < 0.001, ${\text{n}}_{\text{p}}^{2}$ = 0.30 and Social Communication *F*_(3.4, 81.9)_ = 3.40, *p* = 0.017, ${\text{n}}_{\text{p}}^{2}$ = 0.124. As shown in Table 2, participants with high baseline scores showed more robust improvements than their counterparts with middle or low baseline scores. As well, most improvements occurred between baseline and the end of the intervention, with scores showing either stability or more modest improvements at the 3-month follow-up.

Even so, we observed individual differences within these three groups. As such, we also determined the percentage of participants in each group who showed improvements from baseline to the end of the intervention, and from baseline to the 3-month follow-up. Improvements were operationalized as a difference in scores that were at least one-half of each subscale's standard deviation. Summing across subdomains from baseline to the end of the intervention, improvements were found in 25.7% of the low baseline group, 54.8% of the middle group and 84.3% of the high group. Percentages were similar for differences from baseline to the 3-month follow-up (25.6%, 57.0%, and 84.3%, respectively).

### Friendships

#### Number of Friends

As shown in Table 3, over time parents reported significantly more close friends in their offspring with PWS, Cochran's Q (2) = 25.90, *p* < 0.001, with baseline differing from the end of the intervention (*p* < 0.001) and from the 3 month-follow-up (*p* < 0.001). Similarly, participants also reported having more friends over time, Cochran's Q (2) = 12.67, *p* = 0.002. See Table 3. Baseline values differed from the end of intervention (*p* = 0.013) and the 3-month follow-up (*p* = 0.004). At baseline, 55% stated that they had “some” or “lots” of friends but could not specify their names. At the 3-month follow-up, however, these non-specific responses declined to 38.8%, with more individuals naming friends or specifying what they did with them. Further, the number of individuals reporting 0 to 1 friend declined, with a concomitant increase in reporting 2 or more friends, from 8.1 to 38.8%. As participants, and not parents, offered non-specific responses, agreement between them was not assessed.

**Table 3:** Parental- and self-reports of the number of participant's friends over time.

	**Baseline**	**End of intervention**	**3-month follow-up**
**Parental reports**			
0–1	62.7%	45.1%	30.0%
2–3	37.3%	51.0%	58.0%
4 or >	0	3.9%	12.0%
**Participant reports**			
0–1	36.8%	32.6%	22.4%
2–3	6.1%	18.4%	22.4%
4 or >	2.0%	6.2%	16.4%
Non-Specific^+^	55.1%	42.8%	38.8%

+ *Did not specify number or names of friends but stated had “lots” or “some” friends. For Cochran's Q, parental data were dichotomized into 0–1 friend vs. 2–3 and 4 or > friends. For Cochran's Q, self-report data were dichotomized into 1 or more named friends vs. the non-specific category. Analyses did not include the 2 individuals who reported no friends at any time point*.

At Baseline, five individuals indicated that they had no friends, but subsequently named from 1 to 3 new friends at the end of the program or the 3-month follow-up. Two individuals stated that they had no friends at any time point. As all of these seven individuals were no longer in school, follow-up chi-square analyses revealed that participants in vs. out of school were also more apt to report having “some” or “lots” of friends (64% vs. 37.5%, respectively), *X*^2^ (4) = 12.56, *p* = 0.014. No other differences were found in participants who were in or out of school on other outcome variables.

#### Contact With Friends and Getting Along With Others

As summarized in Table 4, parents reported significant increases in the amount of contact that participants had with friends outside of school/work, Cochran's Q (2) = 21.68, *p* < 0.001. Baseline and the end of the intervention differed from the 3-month follow-up (*p* < 0.001 and *p* = 0.014, respectively).

**Table 4:** Parental responses over time regarding frequency of contact with friends and getting along with peers.

	**Baseline**	**End of intervention**	**3-month follow-up**
**How often does your child see friends?**			
< once a week	84.3%	66.7%	47.1%
2–3 times a week	13.7%	29.4%	47.1%
4 or > times a week	2.0%	3.9%	5.8%
**How well does your child get along with Friends/Peers?**			
Worse	49.8%	35.3%	22.4%
Average	46.3%	56.9%	65.8%
Better	3.0%	7.8%	11.8%

*For Cochran's Q, variables were dichotomized into: < one a week vs. 2–3 and 4 or > times a week; and worse vs. average and better*.

No changes were found in how well participants got along with parents or siblings, or when they played or worked alone. A significant improvement, however, emerged in getting along with peers (see Table 4), Cochran's Q (2) = 6.95, *p* = 0.030, with baseline differing from the 3-month follow-up (*p* = 0.016).

#### Coded Descriptions of Friends

Coded responses to “How would you describe a friend?” are summarized in Table 5. Most participants, 49.7%, had 2 codes per response, 33.3% had just 1 code and 17.0% three codes. As no changes over time were found, Table 5 presents the total average percentage across responses. The most frequent codes were positive adjectives (37.7%) and being together and having fun (23.2%). Two codes further reflected how friends benefited participants, being loyal to them (15.2%) and accepting and supportive of them (13.5%). Far fewer individuals, however, described being supportive or loyal to their friends (5.2%). As shown in Table 6, most participants met their friends at school (55%), Special Olympics (11%), and through their family or neighborhood (10%). Table 6 also indicates that participants engaged in a variety of activities with friends.

**Table 5:** Frequency and examples of interview coded responses to “What is a friend?”.

**What is a friend? how would you describe a friend?**		
**Codes**	**%**	**Examples**
Positive adjectives	37.7%	Nice, sweet, kind, funny, polite, friendly, adorable, caring, courteous, cool, energetic, fun
Be together/Have Fun	23.2%	Hang out, talk, have fun, play, stay in touch, do stuff, have conversations, laugh together, have same interests
Trustworthy/Loyal	15.2%	Someone you can trust, tell secrets to, loyal to me, I can rely on, dependable
Supports/Cares for me	13.5%	There for me, helps me out, cares for me, likes me, respects me, nice to me, understands me
Not mean	5.2%	Not take advantage of you, not saying mean things, doesn't bully you, doesn't talk back
Reciprocity	5.2%	You can talk to them and figure out what's wrong and then help them through the tough times; Being kind, respectful and supportive of one another; You stick up for each other and are there for each other in the good times and bad times

**Table 6:** Percentage of interview responses to “What do you like do with your friends for fun?” and “Where did you meet your friends?”.

**What do you like do with your friends for fun?**		**Where did you meet your friends?**	
Go to places, movies	25.7%	School	54.9%
Hang out, talk	25.0%	Special Olympics	10.8%
Play games, other activities	18.6%	Family, Neighbors	9.8%
Physical activities, sports	15.0%	Church	8.8%
Not much	6.1%	Job Training, Work	5.9%
Eat	5.3%	PWS Events	5.9%
Watch TV	4.3%	Camps	3.9%

The majority of respondents projected a positive valence about their friendships. Even so, 26% expressed that they had lost friends (e.g., “I have friends but I call them to hang out with them but they never get back to me cause they're too busy with college and what not”, “Yeah, but I haven't seen them mostly for a long time,” “I have friends from school, but they don't really call me back ‘cause they don't have special needs like I do”). Unsurprisingly, those expressing a loss of friends also reported higher rates of loneliness than others (75% vs. 21.6%), *X*^2^ (1) = 11.39, *p* < 0.001.

### Loneliness

#### Frequency of Loneliness

Table 7 depicts that over time, parents related significantly less loneliness in participants, Cochran's Q (2) = 13.65, *p* =0.001, with Baseline differing from the end of intervention (*p* = 0.001) and the 3-month follow up (*p* < 0.001). Similarly, participants with PWS also reported being less lonely over time, Cochran's Q (2) = 10.43, *p* = 0.005. Baseline values differed from the end of intervention (*p* = 0.009) and from the 3-month follow-up (*p* = 0.003).

**Table 7:** Parental responses to “Complains of loneliness” and participant responses to “Do you ever feel lonely?” over time.

	**Baseline**	**End of intervention**	**3 month follow-up**
**Parental responses**			
Yes	7.8%	3.9%	0
Sometimes	43.2%	27.5%	23.5%
No	49.0%	68.6%	76.5%
**Participant responses**			
Yes	12.2%	6.1%	6.1%
Sometimes	38.8%	28.6%	26.5%
No	49.0%	65.3%	63.4%

*For Cochran's Q, parental and self-reports were dichotomized into no vs. sometimes and yes*.

Although both parents and participants reported diminished loneliness over time, agreement between them was relatively poor. Probing these low kappa's further, increases over time were found in parents and offspring agreeing that they were either lonely or not lonely (baseline agreement = 53.1%, end of intervention = 61.1%, 3-month follow-u*p* = 75.5%). Across the three time points, disagreements occurred in both directions, when parents endorsed loneliness, but participants did not (48.1%) and when participants indicated they were lonely, but parents did not (53.0%).

#### Behaviors if Lonely

When feeling lonely, many (44%) participants reported engaging in activities that distracted them and made them feel better (e.g., listening to music, watching movies), an additional 25% played with their pets. Some (19%) reported that nothing really made them feel better, and just 12% reached out to others.

#### Correlates of Loneliness

Collapsing across all assessments, parental ratings of loneliness were correlated with the CBCL Internalizing domain, *r*_(151)_ = 0.46, *p* < 0.001, specifically with the Anxious/Depressed subdomain, *r*_(151)_ = 0.51, *p* < 0.001. Similarly, participant self-reports of their loneliness were correlated with the Internalizing domain, and Anxious/Depressed subdomain, r's_(151)_ = 0.27 and.31, p's = 0.007, and <0.001, respectively. Comparing correlations between informants using Fisher's r to z transformation confirmed that the strength of these relationships was stronger among parents vs. participants for both the Internalizing domain, *z* = 0.1.89, *p* = 0.03 and Anxious/Depressed subdomain, *z* = 2.01, *p* = 0.019.

## Discussion

This proof-of-concept, mixed-methods study is the first to explore the feasibility and impact of a social skills training program for young people with PWS delivered in an on-line, small group format. The BOSS intervention proved practical and well-tolerated, with excellent participant compliance. Regarding effectiveness, a convergence of findings across informants and methodologies were promising, reflecting improved social skills as well as increased numbers of friends and contact with them, ability to get along with peers and diminished loneliness. No differential effects of clinician vs. student group leaders were found, which bodes well for the implementation of the BOSS curriculum in the broader PWS community.

Over time, significant improvements, with large effect sizes, were found in the SRS's social motivation, communication and cognition subscales. Moreover, on average, participants appeared to maintain their gains in social skills at the 3-month follow-up. Main effects of time, however, were qualified by significant interactions with subscale baseline scores. Those with relatively high baseline SRS scores demonstrated more robust improvements than their counterparts, with 84.3% of this group showing improved scores. Even so, 57% of the middle and 27.4% of the low baseline groups also improved. On the one hand, those entering the intervention with high baselines have more room to improve relative to those that entered with less social dysfunction. Yet, given individual differences across baseline groups, it would be erroneous to conclude that only those who have more social impairments stand to potentially benefit from the BOSS intervention.

The study also included several real-world outcomes that directly bear on the well-being and quality of life for persons with ID, specifically having friends and keeping social isolation and loneliness at bay (3, 17). Regarding friends, both parents and participants reported an increased number of friends, as did participant's naming their friends or specifying what they did with them. Although getting along with family members did not improve, parents reported that participants were getting along better with peers from baseline to the 3-month follow-up. Admittedly, the BOSS curriculum emphasized peer interactions, yet because participants were encouraged to practice specific social skills at home, we had anticipated a possible “spill-over” effect with family members.

Further, from baseline to the 3-month follow-up, parents reported increased contacts with friends. It may be that increased contact with friends was a by-product of learning about and becoming familiar with Zoom as a user-friendly platform to engage with others. It is unclear, however, if increased contact occurred in person, via an online platform or if parents engaged in extra efforts to ensure contact with friends.

Exploring how individuals with PWS meet with friends is especially important as many participants met their friends at school. At baseline, those out of school were more apt to report having no friends, and those in school indicated that they had “a lot” of friends. These findings underscore the importance of post-graduation venues for meeting friends such as Special Olympics, and religious or recreational organizations. As well, Fulford and Cobigo (50) found that adults with ID who were employed were twice as likely to report having friends than those who were unemployed. As only 5 adults in the current study were employed, working or volunteering are also promising avenues for adults with PWS to expand their social networks and make friends.

Participants engaged in a variety of activities with friends, and most described their friendships in positive terms. Even so, 26% noted a loss of friendships, typically with non-disabled peers, and higher rates of feeling lonely than their counterparts. Although friendship loss is not specifically mentioned, Mason et al. (51) found that negative experiences with friends in adults with ID were associated with stress and feelings of vulnerability. Lunsky and Benson (52) reported that distressful social interactions predicted future depressive symptoms and somatic complaints in adults with mild ID. Future studies are needed on the sequalae of both stressful interactions and friendship loss in people with PWS.

As children develop, they move from more egocentric ideas of friends (they do nice things for me or return a favor) to adolescent understandings that friendship involve empathy, mutual trust, reciprocity and shared support (53–55). Despite including these more sophisticated ideas of friends in the BOSS curriculum, participants did not grow in their understandings of what friendships mean. The majority of responses to “How would you describe a friend?” (66.7%) were captured by two or more codes, suggesting that most participants had at least some degree of complexity in conceptualizing friendships. Even so, the majority of participants' responses reflected the positive things that friends provided to them, not necessarily what they provided to their friends. Indeed, only 5.2% identified reciprocity in their views of friendships.

Reciprocity in friendships is associated with the cognitive ability to take another's perspective (54), which as previously noted, is a weakness for many with PWS. As such, future BOSS interventions may need to place more emphasis on these perspective-taking skills. Even so, it is critically important to emphasize the value of friendships at all levels of development in fostering happiness, well-being, psychological adjustment, self-esteem, and learning and refining interpersonal skills (56, 57).

Regarding loneliness, both parents and participants reported reduced loneliness over time, including at the 3-month follow-up. Agreement of loneliness status between participants and parents increased across assessments, from 53.1 to 75.5%. The similar rates of disagreements between informants (when parents, and not participants, endorsed loneliness, and visa versa) raises the question of who is best suited to report on loneliness or other internal states. Given their cognitive and communication challenges, many researchers gather such data from parents or other informants. Yet loneliness is a subjective, internal state, and many have long argued that it best assessed in self versus informant reports, including in those with developmental disabilities (58, 59).

Interventions that reduce loneliness are critically important given the negative sequalae of loneliness on health and mental health. Loneliness in the general population is a potent predictor of such mental health problems as depression, anxiety and suicidal ideation, as well as poor physical health and reduced longevity (60–63). Similar associations between loneliness and mental ill health have been found in people with ID (10). Such relations may be amplified in people with ID as they are at higher risk than the general population for both loneliness and psychiatric, behavioral and emotional problems (10, 64). Heiman (12) found that loneliness was as a significant predictor of depressive symptoms in 310 adolescents with ID. Loneliness was associated with depression in 100 adults with Down syndrome (65), and loneliness in 99 adults with ID was associated with both depression and suicidal ideation (66). Similarly, in the current study, both parental- and self-reported loneliness were correlated with the CBCL's Internalizing domain and Anxious/Depressed subdomain, although such associations were stronger among parents. Further, participants reporting a loss of friends were more likely to report feeling lonely than their counterparts without such losses.

The need for strategies that reduce loneliness in people with PWS or other IDs are magnified by the COVID-19 pandemic. It is well-documented that people across the globe have experienced COVID-19 related spikes in such mental health problems as depression, anxiety, distress, loneliness and anxiety (67, 68). Yet people with ID are especially vulnerable to these and other negative sequalae of social distancing, lockdowns, disrupted daily routines, loss of contact with others, and closures of schools, religious, recreational and other community organizations (69, 70).

Although the BOSS intervention concluded prior to the onset of the COVID-19 pandemic, our research team led informal social groups with individuals with PWS during the first wave of the pandemic. Building on the BOSS curriculum, these informal groups emphasized how participants could connect to one another while also engaging in “good deeds” for group members, their families or communities. In doing so, group leaders stressed the need to adhere to rules (showing respect, common curtesy) and for parental involvement or supervision when individuals decided to form their own online social groups (71). As tele-therapy and other on-line social and behavioral health interventions continue to expand, (72, 73) (REF), future research needs to specify the advantages and disadvantages of these interventions for specific disability groups (74) (REF).

Several study limitations deserve mention. First, as a proof-of-concept study, we did not include a control group, which places limitations on how much we can attribute improvements to the BOSS intervention. We mitigated these limitations by triangulating across different informants and methodologies, with both parents and participants reporting positive effects. Such promising results thus lay the groundwork for further evaluation of the BOSS intervention using a more rigorous, controlled study design.

Second, we did not administer standardized measures of loneliness to participants, opting instead to gather self-reports of loneliness via semi-structured interviews. We did so for two reasons. First, we have found that individuals with PWS in our research programs have difficulty completing standardized questionnaires of their internal states (e.g., anxiety, depression), leading to unreliable data. Second, we have successfully used semi-structured interviews to explore the internal self-representations of young people with PWS (20).

An additional concern is that parental reports of loneliness were based on a single question. Single-item questions are widely used to assess loneliness in the general population (75) yet have met with some controversy. Comparing single- vs. multiple-item measures of loneliness in adults, Mund and colleagues (76) conclude that loneliness can indeed be reliably assessed with single-item questions, including the frequency of feeling lonely.

Relatedly, we did not administer a measure of social network size. Doing so would have added specificity to the types of friendships reported by participants or parents. Although informative, semi-structured interviews do not yield systematic data across individuals. For example, participants may or not offer such details as whether or not their friends also have a disability, if they are in a romantic relationship, or if they counted mentors or care-providers as their friends.

Despite these limitations, this proof-of-concept, mixed-method study justifies future work aimed at improving the social skills of people with PWS. Although challenged by their hyperphagia and food seeking (20), study participants learned social engagement, cognition, communication and motivation skills that furthered their friendships and reduced feelings of loneliness. Further studies are needed, yet findings bode well for the dissemination and implementation of the BOSS curriculum in the broader PWS community.

## Data Availability Statement

The raw data supporting the conclusions of this article will be made available by the authors, without undue reservation.

## Ethics Statement

The studies involving human participants were reviewed and approved by Behavioral Science Institutional Review Board (IRB) at Vanderbilt University. The patients/participants provided their written informed consent to participate in this study.

## Author Contributions

ED conducted statistical analyses, wrote the first draft of the manuscript, and worked with ED and HH-H to devise the intervention curriculum. ER worked with H-HH and ED to devise the intervention curriculum, and also recruited participants, co-led the intervention and trained and supervised graduate students to co-lead the intervention. H-HH worked with ED and ER to devise the intervention, and also recruited participants, co-led the intervention and trained and supervised graduate students to co-lead the intervention. CM conducted inter-rater reliability analyses, entered data, conducted informal social groups, and assisted with data analyses. All authors contributed to the article and approved the submitted version.

## Funding

This research was supported by a grant from the Foundation for Prader-Willi Research entitled Improving social functioning in Prader-willi syndrome.

## Conflict of Interest

The authors declare that the research was conducted in the absence of any commercial or financial relationships that could be construed as a potential conflict of interest.

## Publisher's Note

All claims expressed in this article are solely those of the authors and do not necessarily represent those of their affiliated organizations, or those of the publisher, the editors and the reviewers. Any product that may be evaluated in this article, or claim that may be made by its manufacturer, is not guaranteed or endorsed by the publisher.
